# Use of the Metastatic Lymph Node Ratio to Evaluate the Prognosis of Esophageal Cancer Patients with Node Metastasis Following Radical Esophagectomy

**DOI:** 10.1371/journal.pone.0073446

**Published:** 2013-09-09

**Authors:** Zhenyu He, Sangang Wu, Qun Li, Qin Lin, Junjie Xu

**Affiliations:** 1 State Key Laboratory of Oncology in Southern China, Department of Radiation Oncology, Sun Yat-Sen University Cancer Center, Guangzhou, Guangdong, People’s Republic of China; 2 Guangdong Esophageal Cancer Research Institute, Guangzhou, Guangdong, People’s Republic of China; 3 Xiamen Cancer Center, Department of Radiation Oncology, the First Affiliated Hospital of Xiamen University, Xiamen, Fujian, People’s Republic of China; University of Texas MD Anderson Cancer Center, United States of America

## Abstract

**Objectives:**

The objective of this study was to investigate the number of metastatic lymph nodes (pN) and the metastatic lymph node ratio (MLR) on the post-surgical prognosis of Chinese patients with esophageal cancer (EC) and lymph node metastasis.

**Methods:**

We enrolled 353 patients who received primary curative resection for EC from 1990 to 2003. The association of pN and MLR with 5-year overall survival (OS) was examined by receiver operating characteristic (ROC) and area under the curve (AUC) analysis. The Kaplan-Meier method was used to calculate survival rates, and survival curves were compared with the log-rank test. The Cox model was employed for univariate and multivariate analyses of factors associated with 5-year OS.

**Results:**

The median follow-up time was 41 months, and the 1-, 3- and 5-year OS rates were 71.2%, 30.4%, and 19.5%, respectively. Univariate analysis showed that age, pN stage, and the MLR were prognostic factors for OS. Patients with MLRs less than 0.15, MLRs of 0.15-0.30, and MLRs greater than 0.30 had 5-year OS rates of 30.1%, 17.8%, and 9.5%, respectively (*p* < 0.001). Patients classified as pN1, pN2, and pN3 had 5-year OS rates of 23.7%, 11.4%, and 9.9%, respectively (*p* < 0.001). Multivariate analysis indicated that a high MLR and advanced age were significant and independent risk factors for poor OS. Patients classified as pN2 had significantly worse OS than those classified as pN1 (*p* = 0.022), but those classified as pN3 had similar OS as those classified as pN1 (*p* = 0.166). ROC analysis indicated that MLR (AUC = 0.585, *p* = 0.016) had better predictive value than pN (AUC = 0.565, *p* = 0.068).

**Conclusions:**

The integrated use of MLR and pN may be suitable for evaluation of OS in Chinese patients with EC and positive nodal metastasis after curative resection.

## Introduction

Esophageal cancer (EC) has a higher incidence in China than in most Western countries. Moreover, most cases of EC in China are squamous cell carcinomas, but an increasing percentage of ECs in Western countries are classified as adenocarcinomas [1]. There has been great progress in the comprehensive therapy of EC in recent decades, although the prognosis of EC patients in China remains poor. Radical esophagectomy and subsequent lymph node dissection are the major treatments for EC, but the regimens for postoperative adjuvant therapy vary among different countries [2–5], probably because of geographic differences in the incidence of the different pathological types of EC.

The presence of lymph node metastases affects the prognosis of patients with EC. The first edition of the *Guidelines for TNM Staging* in 1977 staged positive lymph nodes as N1. However, the seventh edition of the American Joint Committee on Cancer (AJCC) *Staging Manual* [6] (published in 2010) reclassified EC lymph node metastases so that stage was based on the number of metastatic lymph nodes. As with breast cancer, gastric cancer, and colon cancer, poor prognosis in EC was associated with a greater number of metastatic lymph nodes. According to current guidelines for EC, pN1 refers to 1-2 positive lymph nodes, N2 refers to 3-6 positive lymph nodes, and N3 refers to 7 or more positive lymph nodes [6].

There is controversy regarding the optimal surgical method for treatment of EC and in the pathological factors to be considered after surgery. Since the publication of the AJCC staging system in 2010, studies of the impact of this new system in Asia indicated an overlap in the survival curves of patients with stage pN2 and pN3 EC [7,8]. This suggests that the new staging system may be not applicable to Asian patients with esophageal squamous cell carcinoma (ESCC) because it does not accurately predict their prognosis and therefore may not provide reliable selection of the most appropriate adjuvant therapy. Several studies in Asia have demonstrated that dissection of a large number of lymph nodes is helpful for accurate staging [9–11]. Current recommendations suggest that at least 18 lymph nodes be dissected [12]. The sixth edition of the AJCC staging system recommended dissection of at least at 7 lymph nodes [13], but this issue was not addressed in the most recent AJCC staging system. Thus, there is uncertainty regarding the number lymph nodes to be dissected, and the current recommendation for dissection of 7 lymph nodes may be insufficient.

The metastatic lymph node ratio (MLR) is the ratio of the number of positive lymph nodes to the total number of dissected lymph nodes. Several studies have confirmed the value of this ratio for evaluation of the prognosis of patients with EC [14–17]. However, few studies have compared the use of the MLR with the new staging system in evaluation of prognosis. In the present study, we retrospectively examined the records of Chinese patients with EC who had lymph node metastasis and received surgical treatment, and compared use of the MLR with the new AJCC staging system for evaluation of prognosis.

## Patients and Methods

### Eligibility Criteria

The study was performed in accordance with the Declaration of Helsinki and was approved by the ethics committee of Sun Yat-Sen University Cancer Center. All patients provided written consent for storage of their information in the hospital database and for use of this information in our research. A total of 353 patients were recruited from Sun Yat-sen University Cancer Center from March 1990 to January 2003. The inclusion criteria were: (i) resectable EC with radical esophagectomy and lymph node dissection; (ii) staging of EC as T1-4 N1-3 M0 according to the seventh edition of the AJCC TNM staging system; (iii) confirmation of squamous cell carcinoma; (iv) negative surgical margin (R0); (v) no pre-surgical radiotherapy or chemotherapy and no post-surgical radiotherapy; (vi) location of EC in the thoracic esophagus.

### Treatment

All patients received radical esophagectomy with primary tumor resection and lymph node dissection. The most common surgical procedures were left thoracotomy, the Ivor-Lewis approach, and the cervico-thoraco-abdominal procedure. The left thoracotomy and the Ivor-Lewis procedure (right thoracotomy) with anastomosis of the upper chest were performed for all tumors of the lower third of the esophagus and some tumors of the middle third. The cervico-thoraco-abdominal procedure was used for all tumors of the upper-third esophagus and some tumors of the middle-third. Extensive lymph node dissection in the posterior mediastinum and abdomen was routinely performed. For lymph node dissection, the thoracic lymphatics were resected through the posterior mediastinum, in other words there was complete dissection of the middle and lower mediastinal nodes, including the perioesophageal, parahiatal, subcarinal, and aortopulmonary window nodes. In the abdominal nodal dissection, the upper abdominal and retroperitoneal lymph nodes were removed, which contained coeliac, splenic, common hepatic, left gastric, lesser curvature, and parahiatal nodes. Clearance of the superior mediastinum was performed. Cervical lymphadenectomy was not routinely performed. For patients with cervical anastomoses, all lymph nodes exposed in the cervical incision were also dissected.

None of the patients received postoperative radiotherapy, and 4.5% of patients received post-surgical chemotherapy (cisplatin and 5-fluorouracil).

### Histopathology of resected lymph nodes

All resected specimens were submitted for pathologic examination. The pathologists read all slides to evaluate the depth of the primary tumors and node involvement, which were separately labeled by the surgeons in a routine manner. One section from each lymph node was analyzed by hematoxylin and eosin (H&E) staining. The nodes that were examined included those that were embedded in the *en bloc* specimen and not labeled by surgeons, but were identified by the pathologists. The lymph node number was counted by low power field microscopy. The total number of resected lymph nodes was the sum of the cervical, intrathoracic, and abdominal lymph nodes. The number of metastatic lymph nodes and the ratio of involved to removed nodes were determined.

Postoperative N staging was according to the seventh edition of the AJCC TNM staging system: pN1, 1-2 metastatic lymph nodes; pN2, 3-6 metastatic lymph nodes; pN3, 7 or more metastatic lymph nodes [6].

### Follow-up

Follow up was performed by hospital visit, telephone, or mail and began on the first day after surgery. The endpoint was overall survival (OS). For patients who died, survival time was determined from the date of surgery to the date of death; for patients who survived, survival was determined from the date of surgery to the date of last follow up.

### Statistical Analysis

Statistical analysis was performed using SPSS version 16.0. The optimum cut-off point for the MLR was determined by use of the receiver operating characteristic (ROC) curve and a step analysis with 0.05 MLR intervals to predict prognosis. The association of pN and MLR with OS was examined by ROC analysis and calculation of the area under the curve (AUC). Calculation of survival rates and univariate survival analysis employed the Kaplan-Meier method, and statistical comparisons employed the log-rank test. Factors with statistically significant differences were included in a Cox proportional hazard model for multivariate analysis. A *p*-value less than 0.05 was considered statistically significant.

## Results

### Univariate analysis of patient characteristics and survival

A total of 353 EC patients (285 males and 68 females) met the inclusion criteria (Table 1). The median age of EC onset was 58 years (range: 25-79 years). There were fewer than 15 dissected lymph nodes in 267 patients, and 15 or more dissected lymph nodes in 86 patients (median: 9 per patient; range: 2-57 per patient). A total of 3925 positive lymph nodes were dissected, and 959 of these were positive (24.4%). There was a median of 2 metastatic lymph nodes per patient (range: 1-20). The median MLR was 0.20 (range: 0.03-1.0).

**Table 1:** Univariate analysis of the association of patient variables with 5-year overall survival (OS) and median survival duration.

**Variable**	**Number**	**5-year OS (%)**	**Median survival (months)**	***P* value**	
**Gender**					
Male	285	18.3	19.5	0.22	
Female	68	25.4	25.2		
**Age**					
≦60	217	22.7	21.6	0.033*	
>60	136	15.1	18.9		
**pT stage**					
T1-2	107	20.3	20.1	0.72	
T3-4	246	19.2	20.2		
**pN stage**					
N1	228	23.7	25.5	<0.001*	
N2	101	11.4	14.9		
N3	24	9.9	11.5		
**MLR**					
<0.15	126	30.1	28.5	<0.001*	
0.15-0.30	105	17.8	20.0		
>0.30	122	9.5	16.8		
**Total dissected lymph nodes**					
<15	267	19	20.7	0.807	
≥15	86	21.8	19.2		
**Tumor location**					
Upper thorax	20	31.3	49.9	0.378	
Middle thorax	195	20.3	23.3		
Lower thorax	138	17.3	18.5		
**Tumor length (cm)**					
≤5cm	119	20.3	20.2	0.894	
>5cm	234	19.1	20.1		
**Tumor differentiation**					
High	80	21.7	19.2	0.822	
Moderate	146	19.8	24.3		
Poor	115	19.3	20.2		
Unknown	12	-	-		
**Postoperative adjuvant treatment**					
Yes	16	15	17.9		0.647
No	337	19.7	20.2		

* *P* value < 0.05 by a log-rank test

The median follow up period was 41 months (range: 0.3-171.6 months). A total of 89 patients survived, 264 patients died, and the median survival time was 20.2 months. The 1-, 3- and 5-year OS rates were 71.2%, 30.4%, and 19.5%, respectively.

### Determination of MLR cut-off points

We initially used ROC analysis with continuous variables and identified the best cut-off point for MLR as 0.15. We then obtained another cut-off point by using step analysis with intervals of 0.05 MLR and the log-rank test for comparisons. The results showed that an MLR cut-off point of 0.30 was significantly associated with poorer prognosis. Thus, we determined cumulative survival rates for patients with MLRs less than 0.15 (n = 126, 35.7%), MLRs between 0.15 and 0.30 (n = 105, 29%), and MLRs greater than 0.30 (n = 122, 34.6%) (Table 1).

### Relationship of MLR and pN with OS

**Figure 1:**
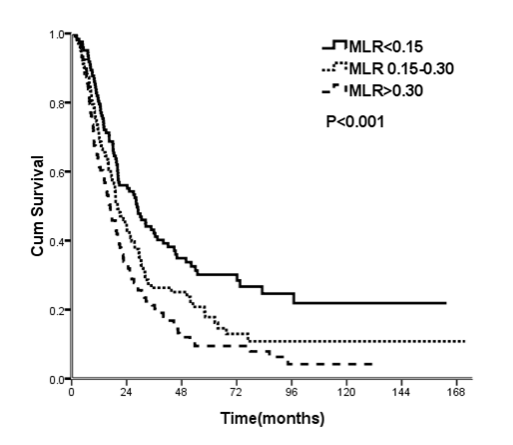
Cumulative survival of patients stratified by metastatic lymph node ratio (MLR).

**Figure 2:**
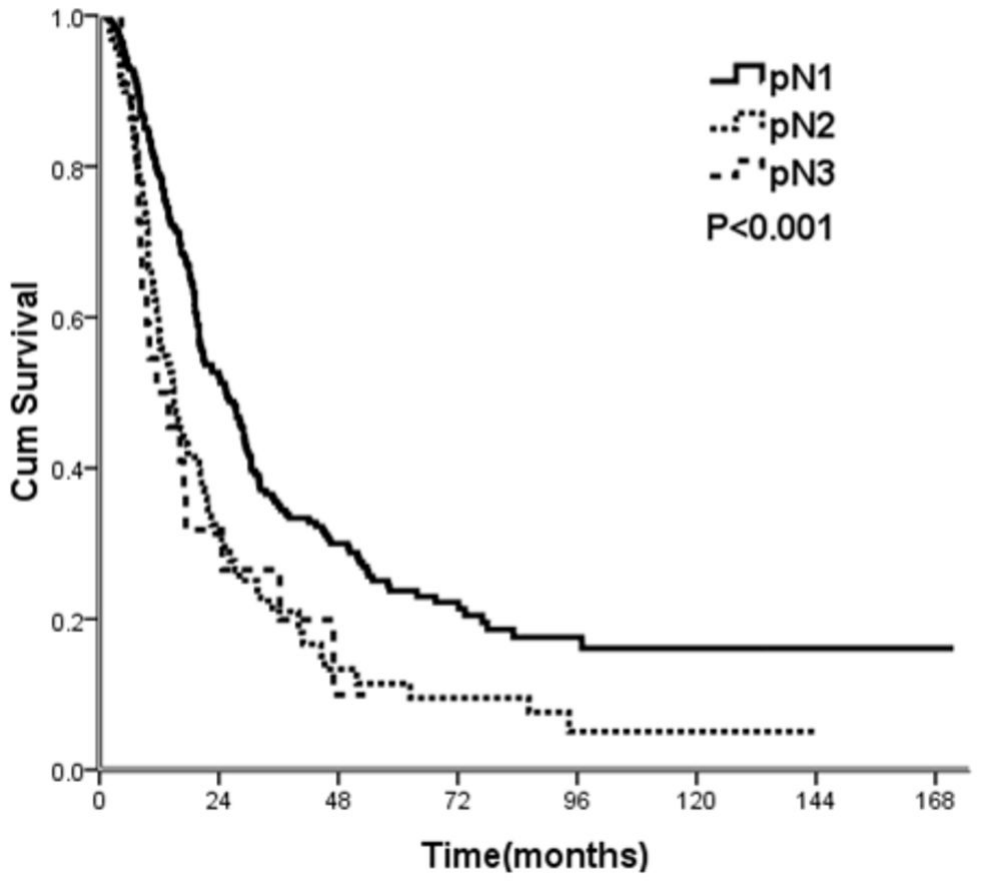
Cumulative survival of patients stratified by the number of lymph node metastases (pN).

Table 1 shows the results of the univariate analysis of patient variables with 5-year OS and median survival. The results show that age, pN stage, and the MLR were significantly associated with OS. Patients with MLRs less than 0.15 had a 5-year OS of 30.1%, those with MLRs of 0.15 to 0.30 had a 5-year OS of 17.8%, and those with MLRs greater than 0.30 had a 5-year OS of 9.5%. The median survival times of these 3 groups were 28.5, 20.0, and 16.8 months, respectively (Figure 1, log-rank test: *p* < 0.001). The results also indicated that pN was associated with OS, but there was significant overlap in the survival curves of patients classified as pN2 and pN3 (Figure 2). For patients classified as pN1, pN2, and pN3, the 5-year OS was 23.7%, 11.4%, and 9.9%, respectively (*p* < 0.001 by a log-rank test). The median survival times of these groups were 25.5, 14.9, and 11.5 months, respectively. Gender, pT stage, EC location, tumor size, degree of differentiation, and postoperative chemotherapy had no influence on OS.

### Multivariate analysis of OS

Multivariate analysis indicated that a greater MLR and advanced age were significantly and independently associated with increased risk for OS. In particular, patients with MLRs less than 0.15 had the best OS, those with MLRs of 0.15 to 0.30 had intermediate OS, and those with MLRs greater than 0.30 had the poorest OS. Patients classified as pN2 had significantly poorer OS than those classified as pN1 (*p* = 0.022), but those classified as pN3 had similar OS as those classified as pN1 (*p* = 0.166) (Table 2).

**Table 2:** Multivariate analysis of the association of patient variables with 5-year overall survival.

**Variable**	***P***	**Hazard ratio**	**95% confidence interval**
**Age**			
>60 vs. ≤60	0.034	1.306	1.021-1.672
**N stage**			
N1		1	Reference
N2	0.022	1.444	1.055-1.977
N3	0.166	1.477	0.851-2.564
**MLR**			
<0.15		1	Reference
0.15-0.30	0.042	1.382	1.012-1.889
>0.30	0.027	1.501	1.047-2.151

### ROC analysis

**Figure 3:**
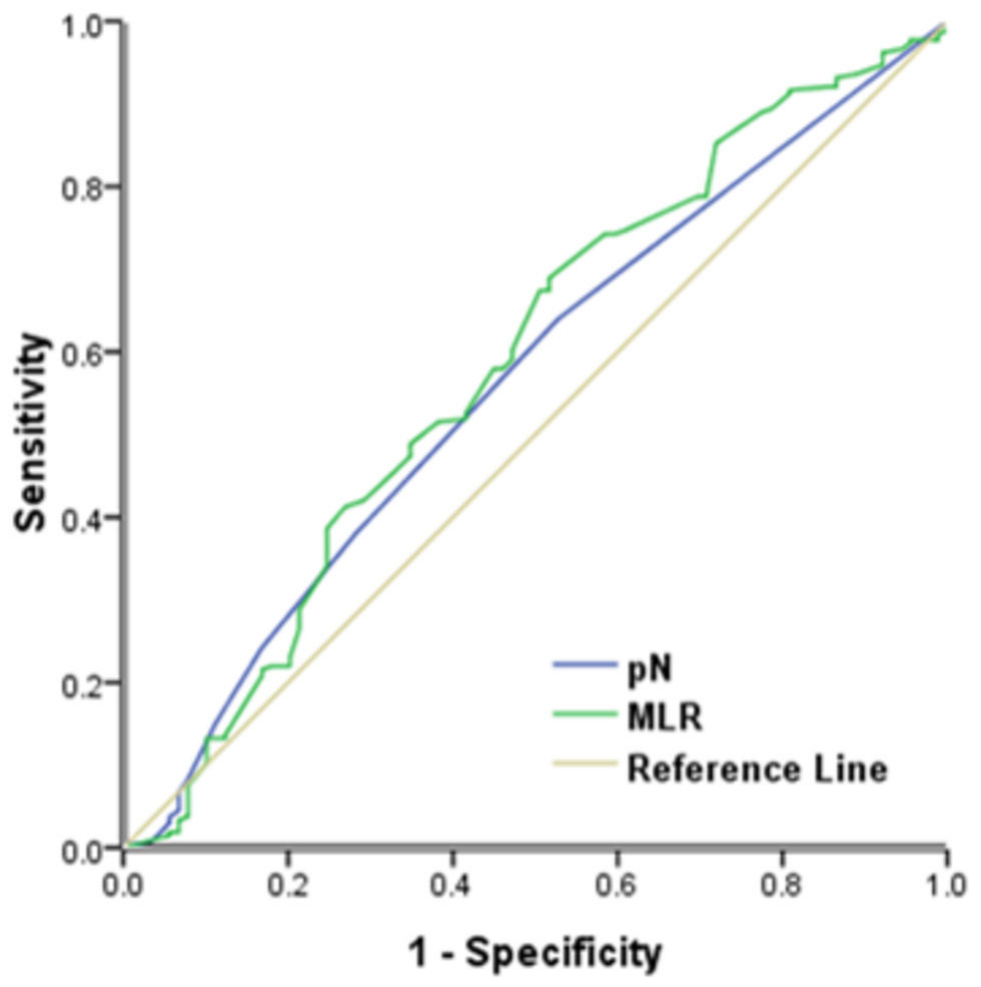
Comparison of receiver operating characteristic curves based on the metastatic lymph node ratio (MLR) and the number of positive lymph nodes (pN).

Finally, we used ROC analysis to analyze the association of pN and the MLR with OS (Figure 3). The results indicate that the MLR (AUC = 0.585, *p* = 0.016, 95%CI: 0.514-0.657) had greater predictive value than pN (AUC = 0.565, *p* = 0.068, 95%CI: 0.496-0.634).

## Discussion

In the present study, we compared the use of pN stage and the MLR for determination of the prognosis of Chinese patients with EC who were positive for lymph node metastasis following radical esophagectomy. The results showed that a high MLR was independently and significantly associated with poor 5-year OS.

The most notable difference between the sixth and seventh editions of the AJCC TMN staging systems for EC is that the sixth system uses quantitative evaluation of metastases in regional lymph nodes. This is believed to provide improved prediction of prognosis for EC patients [6]. However, the surgical methods and the number of dissected lymph nodes vary among patients, so there may be limitations to simply using the number of positive lymph nodes that were detected. The sixth edition AJCC staging system recommends that at least 7 positive lymph nodes be removed [13], but the seventh edition does not have this requirement. Udagawa and Akylyama employed a technique (three-field lymphadenectomy) that allows complete dissection of more than 100 lymph nodes from the lower neck, mediastinum, and upper abdomen in EC patients [18]. Thus, dissection of only a few lymph nodes seems unlikely to provide accurate predictions of prognosis. Improvements are needed in the TNM staging system for EC in order to improve its ability to predict prognosis, so that there can be more appropriate selection of individualized post-surgical therapeutic regimens.

Dissection of a large number of lymph nodes is crucial for accurate staging of EC, but its impact on the determination of prognosis is still controversial. Peyre et al. studied a population of EC patients, 60% of whom had adenocarcinoma, and reported that the number of dissected lymph nodes was an important determinant of prognosis, and that EC patients benefit from the dissection of at least 23 lymph nodes [19]. However, another study of EC patients, 94% of whom had squamous cell carcinoma, indicated that dissection of fewer than 15 lymph nodes rather than 15 or more lymph nodes had no apparent effect on prognosis [14]. The present study of EC patients, all of whom had squamous cell carcinoma, indicated no relationship of the number of dissected lymph nodes with prognosis. Thus, ECs that are classified as squamous cell carcinomas and adenocarcinomas appear to have different characteristics with regard to lymph node drainage.

Recent studies have investigated the influence of the MLR on the prognosis of patients with EC. However, the identification of an MLR cut-off point for accurate prediction of prognosis has not yet been determined. In the present study, we classified patients into 3 groups based on their MLRs, and compared the prognoses of these groups using ROC curve and step analysis at intervals of 0.05 MLR. The results showed patients with MLRs of 0.15 to 0.30 had significantly worse prognosis than those with lower MLRs. Liu et al. found that patients with MLRs greater than 0.50 had a poorer prognosis than those with MLRs of 0.25 to 0.50 and those with MLRs less than 0.25, but there was an overlap in the survival curves (within 3 years) of patients in these two lower MLR groups [17]. Hsu et al. used an MLR of 0.20 as a cut-off, and showed that the 3-year survival rate was 28.7% in patients with MLRs of 0 to 0.20, but 9.8% in those with MLRs greater than 0.20 (*p* < 0.001) [14]. Kelty et al. found that patients with MLRs of 0.01 to 0.19, 0.20 to 0.39, 0.40 to 0.59, and 0.60 and greater had a median survival times of 27.4, 22, 12.8 and 6.5 months (*p* < 0.001) [16]. However, another study of esophageal adenocarcinoma reported no significant differences in survival of patients with MLRs of 0.25 or less, 0.25 to 0.50, and more than 0.50 [15]. These different studies may have used different MLR cut-off points due to differences in the extent of lymph node dissection and the pathological type of EC. Thus, future prospective studies of patients with EC of the same pathological type who received the same surgery and lymph node dissection are required to determine the MLR cut-off value that best correlates with prognosis.

To date, few studies have compared the use of pN stage and the MLR in determination of EC prognosis. Thota et al. [20] studied EC patients, 26.4% with squamous cell carcinoma and 73.6% with adenocarcinoma. Based on the seventh edition AJCC staging system, the 5-year OS rates were 37%, 14%, 5.3%, and 3.4% in patients classified as having stage pN0, pN1, pN2, and pN3, respectively. Moreover, the 5-year OS rates were 35.4%, 7.6%, and 6% in patients with MLRs of 0.0 to 0.1, 0.1 to 0.5, and 0.5 to 1.0, respectively (*p* < 0.0001). However, there was no significant difference in the 5-year OS rates of patients with MLRs of 0.1 to 0.5 and 0.5 to 1.0.

There are well-known differences in the etiology, location, lymph node spread, and prognosis of patients with esophageal squamous cell carcinoma and esophageal adenocarcinoma [21]. Most cases of EC are classified as adenocarcinomas in Western countries, but as squamous cell carcinomas in China and some other countries. This suggests that different staging systems may be needed for China and Western countries. In the present study, the new N staging system did not provide reliable prediction of prognosis in the evaluation of esophageal squamous cell carcinoma in Chinese patients. In particular, there was no significant difference in the 5-year OS of patients with stage pN2 and pN3 EC, possibly because of the small number of patients with pN3 cancer (n = 24, 6.8%). Our univariate analysis of prognosis based on the MLR showed significant differences among our three groups 5-year OS. In addition, multivariate analysis indicated that these differences were statistically independent and significant.

There were some limitations in the present study. First, this was a retrospective study and there was no accurate information on the locations of the lymph node metastasis in the patient records. In future studies, more clinical information should be provided so there can be an assessment of the effect of EC at different sites and of the effect of MLRs at different regions on the prognosis of EC. Second, we were unable to determine the optimal threshold for MLR. Future prospective studies are needed to identify the optimal threshold value of the MLR needed for reliable prediction of prognosis.

In conclusion, our findings demonstrated that consideration of the MLR and the number of metastatic lymph nodes in patients with EC improved predication of the 5-year OS of EC patients and may help in the selection of adjuvant therapy.
